# Integration of Metrology in Grinding and Polishing Processes for Rotationally Symmetrical Aspherical Surfaces with Optimized Material Removal Functions

**DOI:** 10.3390/mi15101276

**Published:** 2024-10-21

**Authors:** Ravi Pratap Singh, Yaolong Chen

**Affiliations:** Department of Mechanical Engineering, Xi’an Jiaotong University; 28 Xianning West Road, Xi’an 710049, China

**Keywords:** metrology, quality control, material removal function, error compensation, CAM software

## Abstract

Aspherical surfaces, with their varying curvature, minimize aberrations and enhance clarity, making them essential in optics, aerospace, medical devices, and telecommunications. However, manufacturing these surfaces is challenging because of systematic errors in CNC equipment, tool wear, measurement inaccuracies, and environmental disturbances. These issues necessitate precise error compensation to achieve the desired surface shape. Traditional methods for spherical optics are inadequate for aspherical components, making accurate surface shape error detection and compensation crucial. This study integrates advanced metrology with optimized material removal functions in the grinding and polishing processes. By combining numerical control technology, computer technology, and data analysis, we developed CAM software (version 1) tailored for aspherical surfaces. This software uses a compensation correction algorithm to process error data and generate NC programs for machining. Our approach automates and digitizes the grinding and polishing process, improving efficiency and surface accuracy. This advancement enables high-precision mass production of rotationally symmetrical aspherical optical components, addressing existing manufacturing challenges and enhancing optical system performance.

## 1. Introduction

In modern society, optical components are integral to a wide range of industries, including optics, aerospace, medical devices, and telecommunications [1,2]. These components typically encompass planes, spheres, and, more recently, aspherical surfaces. The refractive index and direction of incident light differ across these types, leading to variations in imaging effects. Planar and spherical optical elements, with their straightforward geometries, can achieve high surface accuracy through traditional machining techniques, such as grinding, polishing, lapping, centering, diamond turning, and sometimes chemical etching [3]. However, despite their high yield and ease of mass production, spherical optical systems inherently suffer from optical aberrations such as low definition and peripheral distortion due to the differing focal points for off-axis rays. To mitigate these aberrations, traditional optical designs often employ multiple spherical mirrors with varying radii of curvature [4]. While this can improve imaging quality, it also results in more complex and costly optical systems.

In contrast, aspheric lenses, characterized by their non-spherical surfaces, represent a significant advancement in optical technology [5]. The inclusion of higher-order curvature allows for independent correction of spherical aberration, leading to more efficient and simpler optical systems [6]. Aspherical surfaces have the ability to focus light more accurately, improving the performance of telescopes and satellite imaging systems to achieve high-resolution images of space and the Earth’s surface. These properties make them indispensable for space exploration and Earth observation missions [7]. The automotive industry also benefits significantly from aspherical optics. Modern vehicles utilize aspherical lenses in headlights and rear-view mirrors to improve driver visibility and safety. Aspherical headlights provide better illumination patterns, reducing glare for oncoming traffic and enhancing night-time driving conditions. In rear-view mirrors, aspherical designs reduce blind spots, offering a wider field of view and improving overall vehicle safety [8]. In the medical field, aspherical lenses and mirrors are used in a variety of diagnostic and therapeutic devices. For instance, in endoscopy, aspherical lenses provide clearer and more detailed images of internal organs, aiding in accurate diagnosis and treatment. Ophthalmic devices, such as corrective eyeglass lenses and contact lenses, also benefit from aspherical designs to improve vision correction by minimizing distortions [9]. The telecommunications industry employs aspherical lenses in fiber optics to improve signal clarity and transmission efficiency. Aspherical components help focus light precisely on optical fibers, reducing signal loss and enhancing the performance of communication networks. This precision is critical for high-speed data transmission and reliable internet connectivity [10].

Despite the numerous advantages and broad applications of aspherical surfaces, their manufacturing presents significant challenges. The complex geometry of aspherical surfaces, with their varying curvature, demands exceptional precision during production [11]. Several key difficulties arise in achieving the high precision required for these surfaces [12]. The production process is highly susceptible to systematic errors in CNC (Computer Numerical Control) equipment. These errors can result from inaccuracies in machine calibration, misalignment of components, and limitations in the control algorithms. Such inaccuracies are amplified when dealing with the intricate shapes of aspherical surfaces, leading to deviations from the desired surface profile [13]. Tool wear is a critical factor affecting precision. During the grinding and polishing processes, tools undergo wear and tear, altering their shapes and effectiveness. This wear is not uniform and can lead to uneven material removal, introducing errors in the surface geometry. Continuous monitoring and compensation for tool wear are essential to maintain precision, yet this adds complexity to the manufacturing process [14]. To monitor tool wear effectively and ensure the desired surface quality, various methods and devices are employed. Advanced microscopy techniques play a pivotal role in this regard. For example, 3D focus variation microscopes utilize multiple focal planes to construct a detailed 3D profile of the surface, enabling the identification of wear patterns that may not be visible through traditional methods. Interferometric microscopy offers high-resolution surface measurements by analyzing interference patterns created by the interaction of light waves, allowing for the detection of minute changes in surface geometry due to wear [15]. Confocal microscopy is another valuable technique, providing optical sectioning capabilities to generate high-resolution images. This method is particularly useful for examining surface roughness and detecting wear at various depths, thereby contributing to a more comprehensive understanding of tool performance over time [16]. In addition to optical methods, elastomeric tactile sensors are gaining traction in wear measurement. These sensors can conform to the surface profile and directly measure wear by assessing changes in surface texture and roughness. Their flexibility allows for real-time monitoring during the manufacturing process, providing immediate feedback on tool performance and wear characteristics [17].

Measurement inaccuracies of aspherical surfaces also pose significant challenges. Accurate measurement of aspherical surfaces is difficult because of their non-uniform curvature [18]. Traditional measurement techniques used for spherical and planar surfaces are inadequate for aspherical geometries [19]. Advanced metrology tools are required to capture the surface profile precisely, but these tools themselves are susceptible to calibration errors and environmental influences such as temperature fluctuations and vibrations [20]. Environmental disturbances further complicate the production process. Variations in temperature, humidity, and vibrations can affect both the manufacturing equipment and the material being processed [21]. Such disturbances can lead to thermal expansion or contraction of the materials, misalignment of equipment, and other issues that compromise the precision of the finished aspherical surface [22].

To address these challenges, precise error compensation strategies are necessary. Traditional methods used for spherical optics do not suffice for aspherical components, necessitating the development of specialized approaches. This study aims to tackle these issues by integrating advanced metrology with optimized material removal functions in the grinding and polishing processes. Through the combination of numerical control technology, computer technology, and data analysis, we developed CAM software specifically designed for aspherical surfaces. This software employs a compensation correction algorithm to process error data and generate accurate NC programs for machining, thereby automating and digitizing the grinding and polishing process.

## 2. Analysis of Material Removal Mechanism and Its Coordinate System

### 2.1. Material Removal Mechanism in Grinding

**Figure 1:**
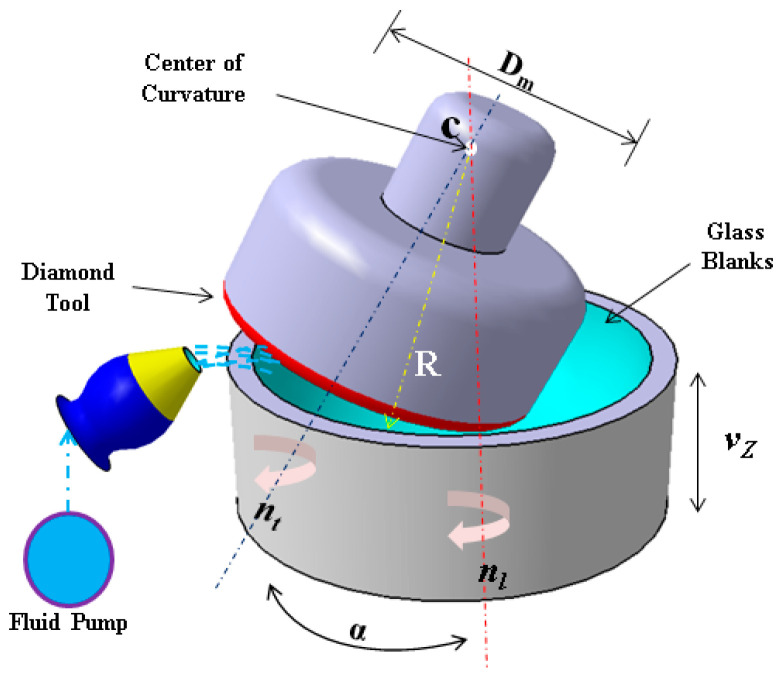
Schematic diagram of the grinding process.

Grinding is a crucial process in the production of aspherical surfaces, characterized by high precision and material removal rates. The fundamental principle involves oblique cutting, where the contact path between the workpiece and the diamond cutting tool forms a circular trajectory during machining. This circular plane is angled relative to the workpiece axis, creating what is known as a truncated circle. In this method, variations in error have minimal impact on the surface quality, allowing for high-precision surfaces to be achieved with fewer coordinate variables. The schematic diagram of the generating process is shown in Figure 1. The formula for calculating the processing angle is given as

(1) $$\sin\alpha =\frac{D_{m}}{2\left(R\pm r\right)}$$

where *D_m_* = diamond grinding wheel pitch diameter, *R* = machined spherical radius, *r* = diamond grinding wheel end arc, and *α* = axis swing angle.

### 2.2. Material Removal Mechanism in Polishing

**Figure 2:**
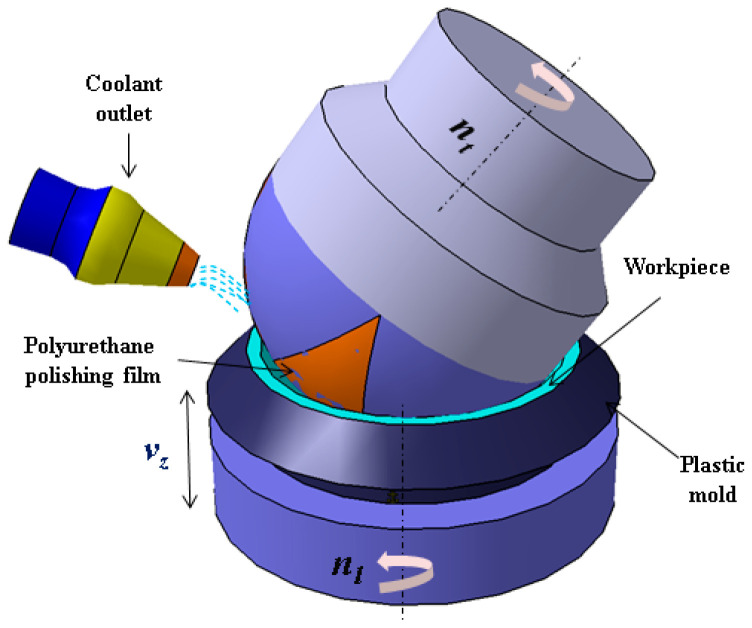
Schematic diagram of the polishing process.

The polishing process of an aspherical lens begins with grinding to shape the lens surface into a preform close to the desired aspherical profile. This preform still contains irregularities and inaccuracies that must be eliminated by polishing to achieve the final precision optics. Figure 2 illustrates a schematic diagram of the polishing process.

Modeling the entire polishing process mathematically and physically is challenging because of its complexity. Factors like temperature, slurry concentration, particle size, pH value, workpiece speed, and feed rate all influence material removal rates. Consequently, creating an accurate mathematical relationship between all polishing parameters is nearly impossible. To address these challenges, researchers have developed mathematical models with certain assumptions. One of the most notable is the Preston equation, proposed by F.W. Preston in 1927. The Preston equation describes the CNC polishing process as a linear relationship over a broad numerical range, suggesting that the material removal rate is proportional to the pressure applied by the polishing pad and the relative velocity between the pad and the workpiece. The mathematical model for the amount of material removed during polishing per unit time is given by [23].

(2) $$\frac{dZ}{dx}=KV\left(x,y,t\right)P\left(x,y,t\right)$$

where d*Z*/d*x*—the amount of material removed per unit time; *K*—Preston constant, with polishing die material, workpiece material, polishing liquid concentration, temperature, and other factors; *V*(*x*, *y*, *t*)—the relative speed of the polishing head and the workpiece at the point of contact degree; and *P*(*x*, *y*, *t*)—the instantaneous pressure between the polishing head and the workpiece at the point of contact.

### 2.3. Coordinate System for Describing Rotationally Symmetrical Aspheric Surfaces

**Figure 3:**
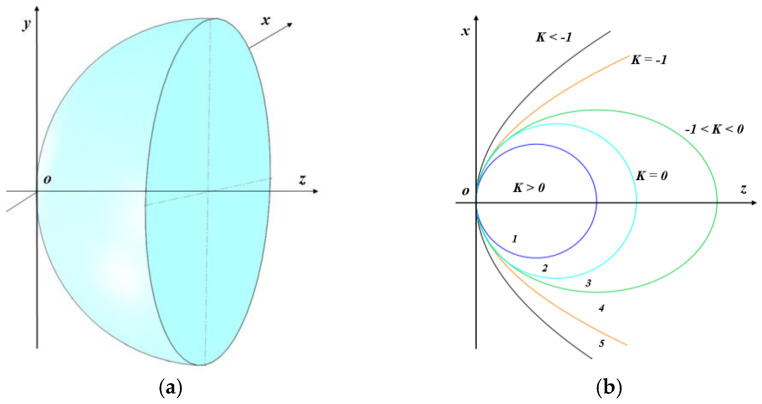
Cartesian coordinate system of aspheric surfaces. (**a**) Aspheric surface. (**b**) Quadratic curve for with K.

Manufacturing rotationally symmetrical aspheric surfaces poses significant challenges because of their complex shapes. Currently, the standard right-hand Cartesian coordinate system is used internationally to describe aspheric surfaces, as shown in Figure 3a. The coordinate system is configured with the vertex of the aspheric surface positioned at the origin. The *z*-axis serves as the optical axis, oriented from left to right.

Aspheric surfaces are typically described using meridian cross-section curves. In this setup, the *xoz* coordinate plane represents the plane of the meridian section. For any point on an aspheric surface, the radius of curvature is determined by the position of the center of curvature relative to the vertex. Specifically, if the center of curvature is to the right of the vertex, the radius of curvature is positive. If the center of curvature is to the left of the vertex, the radius of curvature is negative. This convention ensures a consistent and precise description of aspheric surfaces, facilitating accurate design and analysis in optical applications.

The equation for the meridian section curve of an aspheric surface is typically composed of the following parts: a datum quadric surface and an additional polynomial. The additional polynomials are generally expressed as polynomials of even power series. The equation can be written as

(3) $$z\left(x\right)=\frac{x^{2}}{R_{0}\left[1+\sqrt{1-\left(1+K\right){\left(x/R_{0}\right)}^{2}}\right]}+\sum_{i=1}^{n}A_{i}x^{2i+2},x\in \left[0,\frac{\Phi_{0}}{2}\right]$$

where *z*(*x*) represents the height of the surface along the z-axis, *R*_0_ is the radius of curvature at the vertex of the aspheric curve, defined as *R*_0_ *=* 1/*c*, where *c* is the curvature at the apex of the aspheric curve, Φ_0_ is the clear aperture of the workpiece to be processed, *K* is the quadratic coefficient (with *K=* −*e*^2^), *e* is the eccentricity of the aspheric surface, and *A_i_* (where *i =* 1, 2, *… n*) denotes the surface coefficients of the higher order terms of the spheric surface.

Because of the nature of rotational symmetry, the range of values for *x* is desirable as non-negative fractions. The quadratic coefficient *K* varies based on the quadratic curve types, as shown in Figure 3b. The radius of curvature at each point on the rotationally symmetrical aspheric surface is different, and it can be calculated as

(4) $$R\left(x\right)=\frac{{\left[1+{\left(\frac{dz}{dx}\right)}^{2}\right]}^{\frac{3}{2}}}{\left|\frac{d^{2}z}{dx^{2}}\right|}$$

where d*z*/d*x* refers to the first derivative of the meridian section equation at any point on the surface of the workpiece, and d^2^*z*/d*x*^2^ refers to the second derivative of the surface equation at any point on the workpiece.

**Figure 4:**
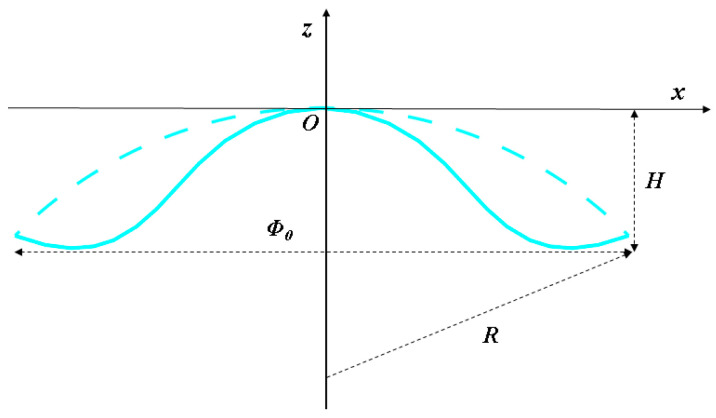
Schematic diagram of the best reference sphere.

In the material removal process of aspheric components, the spherical profile is first machined, which differs from the aspherical profile. The difference between the corresponding points in the x-direction of the surface profile in the z-direction is the asphericity, and the maximum difference is the maximum asphericity $A_{\max}^{0}$. The sphere with the minimum value of maximum asphericity is called the best reference sphere. A schematic diagram of the best reference sphere is given in Figure 4.

The maximum asphericity $A_{\max}^{0}$ can be calculated as

(5) $$\begin{array}{l} R=\frac{\Phi_{0}^{2}+4H^{2}}{8H}\\ A_{\max}^{0}=-\frac{K\Phi_{0}^{4}}{512R^{3}} \end{array}$$

where Φ_0_ is the diameter of the workpiece and *H* is the height. From Equation (5), the maximum degree of asphericity can be achieved when $x=\Phi_{0}/2\sqrt{2}$.

## 3. Establishment of the Optimized Material Removal Model

### 3.1. Mathematical Model of Material Removal

In the process of material removal, the finished workpiece surface is typically divided into numerous small areas to determine the material removal amount on the surface. The material removal amount for each differential area is then calculated, and the overall removal distribution is accumulated. This method requires extensive calculations, so an idealized assumption is proposed to simplify the process. Firstly, considering the physical model, material removal on the workpiece surface occurs during both grinding and polishing. During grinding, large amounts of material are removed to form the rough shape of the workpiece. In polishing, the feed speed of the polishing head is significantly lower than its rotation speed. Therefore, the material removal by the polishing head per unit time can be analyzed based solely on the movement of the polishing head, neglecting the minor error influence caused by its feed.

Secondly, from a mathematical perspective, maintaining a constant material removal rate by the polishing head is challenging. Generally, physical and chemical reactions occur during polishing, causing surface distortion of the workpiece. Without using fluid non-contact polishing or a flexible polymer polishing head, it is impossible to ensure constant pressure at each point of contact. Additionally, considering the edge effect, when the polishing head moves to the edge of the workpiece, the contact width between the polishing head and the workpiece changes, affecting the relative contact pressure and thus altering the material removal rate. However, if the polishing head is not exposed, the relative contact pressure remains constant, and the rotation speed is relatively low, making it feasible to consider the material removal rate constant.

**Figure 5:**
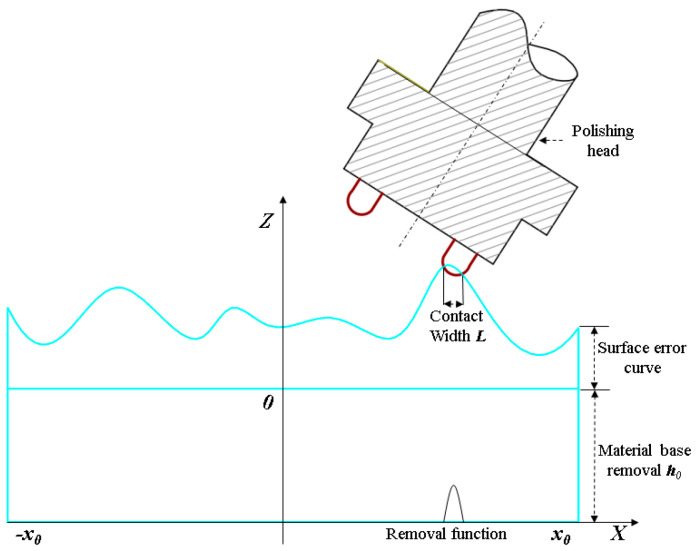
Mathematical model of material removal.

The mathematical model of polishing material removal is illustrated in Figure 5. In the equal compression polishing process, the contact width between the polishing head and the workpiece remains constant, denoted as *L*, and the workpiece diameter is *2x*_0_. The polishing process aims to reduce material continually, ideally polishing all materials to the lowest point of error based on the given surface error data. Considering residence time *D*(*x*, *y*) as an independent variable, residence time in the polishing process is given as

(6) $$T=\int_{-x_{0}}^{x_{0}}\int_{0}^{L}D\left(x,y\right)dxdy$$

where *D*(*x*, *y*) is the residence time of the polishing head, and *T* is the total residence time of the polishing process. When the polishing head does not move, the average removal amount per unit time *R*(*x*, *y*) is given as

(7) $$R\left(x,y\right)=\underset{T\to \infty }{\lim}\left[\frac{1}{T}\int_{-x_{0}}^{x_{0}}\Delta h\left(x,y\right)dt\right]$$

If the removal function applies to any polishing area, the total amount of material removed from the workpiece surface is the sum of removal amounts at each point. Thus, the removal function of the polishing head can be denoted as *δα* and the residence time function of the workpiece surface as *δβ*. By superimposing numerous elements over the polishing head path, the material removal expression can be derived as follows:

(8) $$\Delta h\left(x,y\right)=\underset{\alpha \to 0,\beta \to 0}{\lim}\sum_{\alpha }\sum_{\beta }R\left(x-\alpha ,y-\beta \right)D\left(\alpha ,\beta \right)\delta \alpha \delta \beta$$

When *δαδβ* approaches 0, *δαδβ* can be infinitely reduced to the area element *dαdβ*, and then there are

(9) $$\Delta h\left(x,y\right)=\int_{\alpha }\int_{\beta }\left[R\left(x-\alpha ,y-\beta \right)D\left(\alpha ,\beta \right)d\alpha d\beta \right]$$

The total material removed from the workpiece is expressed as a two-dimensional convolution of the removal function *R*(*x*, *y*) and the residence time *D*(*x*, *y*) as follows:

(10) H(x,y)=R(x,y)∗D(x,y)

After polishing, the residual error *E*(*x*, *y*) can be calculated as

(11) E(x,y)=H(x,y)−R(x,y)∗D(x,y)

### 3.2. Equivalent Material Removal Model of Aspheric Polishing

**Figure 6:**
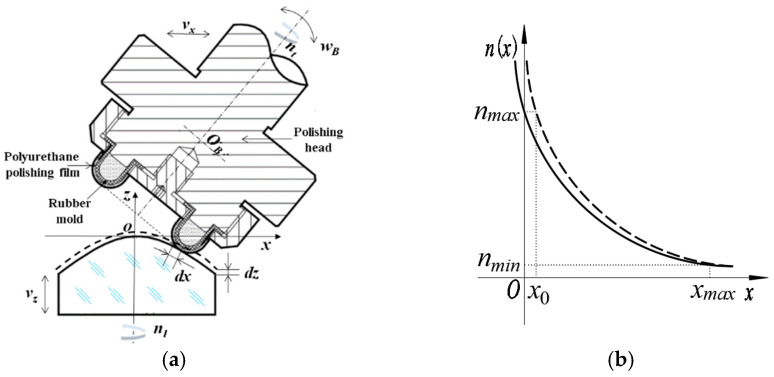
Schematic diagram of the equivalent model of material removal. (**a**) Equivalent material removal. (**b**) Relation between workpiece rotation speed and machining position.

A schematic diagram of equivalent material removal model of aspheric polishing is given in Figure 6. *xoz* is the workpiece coordinate system, the origin of the coordinate system coincides with the vertex of the workpiece surface, and *p*(*x*, *z*) is any point on the workpiece.

During a small unit of time, the polishing head moves a tiny distance d*x* in the x-direction and the workpiece shifts by d*z* in the z-direction. As the workpiece rotates at speed *n*(*x*), the polishing head effectively removes material in a cylindrical shape on the workpiece surface. This cylindrical volume has a length of *2πxn*(*x*), a width of d*z*, and a height of d*x*. This process can be described as

(12) dV=2πxn(x)dxdz

Consider two points *p*_1_(*x*_1_, *y*_1_) and *p*_2_(*x*_2_, *y*_2_) at different positions on the workpiece. To ensure that the material removal per unit time is the same, it is necessary to satisfy,

(13) $$2\pi x_{1}n\left(x_{1}\right)dxdz=2\pi x_{2}n\left(x_{2}\right)dhdx$$

Equation (13) can be reduced to

(14) xn(x)=Const

From the relationship between the workpiece rotation speed and machining position in Figure 6b, when the polishing head reaches position *x*→0, the theoretical rotation speed *n*(*x*)*→∞.* However, in practical machining, the workpiece rotation speed cannot actually reach infinity. Therefore, the maximum rotation speed of the workpiece at the coordinate origin, *x = x*_0_, represents a practical limit. To find the actual rotation speed curve, we shift the theoretical rotation speed curve leftward by *x*_0_. Here, the solid line depicts the actual rotation speed curve, while the dotted line represents the theoretical one. From here, Equation (14) can be written as

(15) $$\left(x+x_{0}\right)n\left(x+x_{0}\right)=Const$$

During numerical control polishing, the workpiece rotational speed reaches its maximum *n*_max_ at the center *x* = 0 and its minimum *n*_min_ at the edge *x = ϕ*_0_/2. Based on these two points, we obtain

(16) $$\{\begin{array}{l} x_{0}n_{\max}=x_{\max}n_{\min}=Const\\ x_{\max}-x_{0}=\frac{\phi }{2} \end{array}$$

From Equation (16), we obtain

(17) $$x_{0}=\frac{\phi n_{\min}}{2\left(n_{\max}-n_{\min}\right)}$$

Combining Equations (15)–(17), the rotational speed at each machining position on the surface of the workpiece is given as

(18) $$n\left(x\right)=\frac{\phi n_{\max}n_{\min}}{\phi n_{\min}+2x\left(n_{\max}-n_{\min}\right)}$$

From Equation (18), we observe that once the max and min speeds of the workpiece are established, the rotational speed at any point *p*(*x*, *z*) on the workpiece surface can be uniquely determined. During polishing, since there is less material at the center and more at the edges of the workpiece, ensuring consistent material removal across all points requires varying processing times at different points. To synchronize the amount of material removed at each point, the feed speed *F*(*x*) of the polishing head must be adjusted relative to the rotational speed *n*(*x*) of the workpiece. This relationship is expressed as follows when the polishing head advances by a step Δ*x*:

(19) F(x)=n(x)·Δx

When the processing feed step is set, the feed speed of the polishing head is directly proportional to the workpiece speed. This means the head feed rate depends on the machining position. The feed speed at each processing point on the workpiece surface can be obtained by combining Equations (18) and (19)

(20) $$F\left(x\right)=\frac{\phi n_{\max}n_{\min}\Delta x}{\phi n_{\min}+2x\left(n_{\max}-n_{\min}\right)}$$

### 3.3. Calculation of the Feature Removal Amount

**Figure 7:**
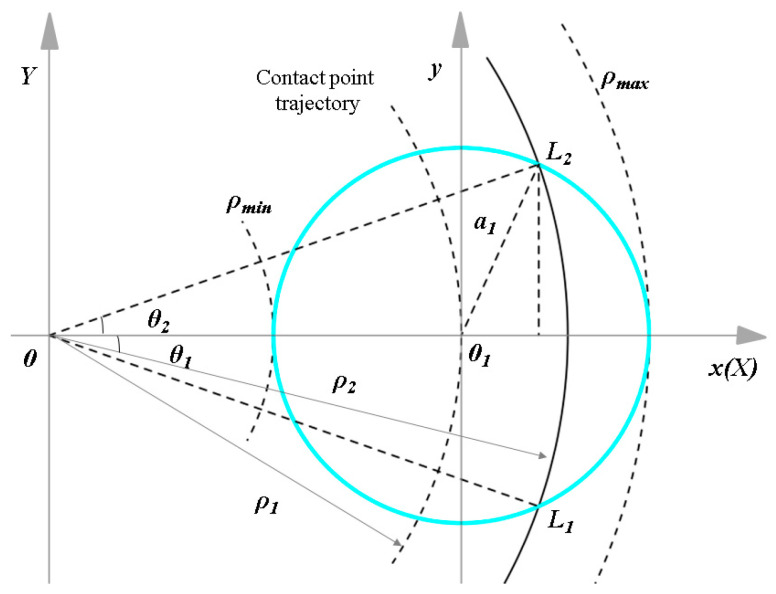
Schematic diagram of material removal during aspheric motion polishing.

A schematic diagram of the material removal during aspheric motion polishing is shown in Figure 7. In the coordinate system *OXY*, the contact area is wide. If the degree is a, then *ρ*_2*max*_ *= ρ*_1_ *+ a*, *ρ*_2*min*_ *= ρ*_1*−a*_, and the angle range corresponding to the arc *l*_1_*l*_2_, is [*ϴ*_1_, *ϴ*_2_]. Here, *ϴ*_1_, *ϴ*_2_ can be calculated as

(21) $$\theta_{2}=\arccos\left(\frac{\rho_{1}^{2}+\rho_{2}^{2}-a^{2}}{2\rho_{1}\rho_{2}}\right),\theta_{1}=-\arccos\left(\frac{\rho_{1}^{2}+\rho_{2}^{2}-a^{2}}{2\rho_{1}\rho_{2}}\right)$$

The residence time d*t* of the polishing head on the workpiece surface is proportional to the length of the polishing track d*l*, while the feed of the polishing head is inversely proportional to the length of the polishing track d*l*. This relationship can be expressed as

(22) $$dt=\frac{dl}{F}=\frac{\rho d\theta }{F}$$

Combining Equations (2) and (22), we obtain

(23) $$\frac{dh\left(x,y\right)}{dl}=\frac{KP\left(x,y\right)V\left(x,y\right)}{F}$$

When converted to polar coordinates, we obtain

(24) $$\frac{dh\left(\rho ,\theta \right)}{dl}=\frac{KP\left(\rho ,\theta \right)V\left(\rho ,\theta \right)\rho }{F}$$

When the polishing head is fed in the negative direction of the x-axis, the feed speed in the contact area also moves negatively along the x-axis, increasing from *ρ*_2*min*_ to *ρ*_2*max*_. This indicates there is

(25) $$F=\frac{\rho }{\rho_{1}}F_{0}$$

Combining Equations (24) and (25), we obtain

(26) $$h\left(\rho ,\theta \right)=\frac{K\rho_{1}}{F_{0}}\int_{\theta_{1}}^{\theta_{2}}P\left(\rho ,\theta \right)V\left(\rho ,\theta \right)d\theta$$

Using polar coordinates to indicate the pressure distribution in the contact area

(27) $$P\left(\rho ,\theta \right)=P_{0}\sqrt{1-\frac{{\left(\rho_{2}\cos\theta \;-\rho_{1}\right)}^{2}+\;{\left(\rho_{2}\sin\theta \right)}^{2}}{a^{2}}}$$

By substituting Equations (18) and (27) in Equation (27), we obtain the expression of feature material removal amount

(28) $$h\left(\rho ,\theta \right)=\frac{3K\rho_{1}P}{F_{0}a^{3}}\int_{\theta_{1}}^{\theta_{2}}\sqrt{\left(a^{2}+\rho_{1}^{2}+\rho_{2}^{2}-2\rho_{1}\rho_{2}\cos\theta \right)}*\sqrt{\left[\begin{array}{l} {\left(n_{T}\rho_{1}\right)}^{2}+{\left(n_{T}+\frac{\phi n_{\max}n_{\min}}{\phi n_{\min}\;+\;2x\left(n_{\max}-n_{\min}\right)}\right)}^{2}\rho_{2}^{2}-\\ 2\rho_{1}\rho_{2}n_{T}\left(n_{T}+\frac{\phi n_{\max}n_{\min}}{\phi n_{\min}\;+\;2x\left(n_{\max}-n_{\min}\right)}\right)\cos\theta \end{array}\right]d\theta }$$

### 3.4. Pressure Distribution Model in the Contact Area

In aspheric polishing, the polishing film is typically softer than the aspheric components, allowing the polishing head and workpiece surface to establish adaptive contact. This adaptive contact ensures effective engagement as the polishing head feeds across different curvature points of the workpiece surface. During the polishing process, the polishing head applies a normal force to the workpiece surface, creating a small contact area where material removal occurs because of the elastic deformation of both the polishing head and the workpiece surface. The pressure distribution in this contact area is often analyzed using Hertz contact theory, which describes how compressive stress is distributed when two elastic bodies are in contact.

Given that the radius of the polishing head edge is much smaller than the radius of curvature of the non-spherical surface, their contact can be approximated as surface-to-surface contact. This results in the formation of a small elliptical contact region when the surfaces come into contact and compress each other. The size and shape of this contact area, as well as the pressure distribution, vary with changes in the polishing head size and the workpiece curvature radius. The ellipse contact area can be expressed as

(29) $$\frac{x^{2}}{a^{2}}+\frac{y^{2}}{b^{2}}=1$$

where *a* is the long half-axis of the ellipse and *b* is the short of the ellipse. *a* and *b* are expressed as

(30) $$\left\{ \begin{array}{l} a=m^{3}\sqrt{\frac{3F}{4A}\left(\frac{1-\mu_{1}^{2}}{E_{1}}+\frac{1-\mu_{2}^{2}}{E_{2}}\right)}\\ b=n^{3}\sqrt{\frac{3F}{4A}\left(\frac{1-\mu_{1}^{2}}{E_{1}}+\frac{1-\mu_{2}^{2}}{E_{2}}\right)} \end{array}\right.$$

where *F* is the normal force of the polishing head on the workpiece; *µ*_1_ and *µ*_2_ are the Poisson’s ratio of the polishing film and workpiece material, and *E*_1_ and *E*_2_ are the modulus of elasticity of the polishing film and workpiece material, respectively. Now, we denote *p*_0_ as the maximum compressive stress in the contact area and *P* as the maximum pressure

(31) $$P=\int \int pdA=\frac{2}{3}\pi abp_{0}$$

From Equation (30), we obtain

(32) $$p_{0}=\frac{3P}{2\pi ab}$$

Therefore, the pressure distribution in the elliptical contact region can be expressed as

(33) $$p\left(x,y\right)=p_{0}\sqrt{1-\frac{x^{2}}{a^{2}}-\frac{y^{2}}{b^{2}}}$$

By substituting the value of *a* and *b* in Equation (33), the theoretical pressure distribution in the elliptical contact region can be obtained as

(34) $$\begin{array}{l} P=\frac{3F}{2\pi mn{\left(\frac{3F}{4A}\left(\frac{1-\mu_{1}^{2}}{E_{1}}+\frac{1-\mu_{2}^{2}}{E_{2}}\right)\right)}^{\frac{2}{3}}}*\\ \sqrt{1-\frac{x^{2}}{m^{2}{\left(\frac{3F}{4A}\left(\frac{1-\mu_{1}^{2}}{E_{1}}+\frac{1-\mu_{2}^{2}}{E_{2}}\right)\right)}^{\frac{2}{3}}}-\frac{y^{2}}{n^{2}{\left(\frac{3F}{4A}\left(\frac{1-\mu_{1}^{2}}{E_{1}}+\frac{1-\mu_{2}^{2}}{E_{2}}\right)\right)}^{\frac{2}{3}}}} \end{array}$$

**Figure 8:**
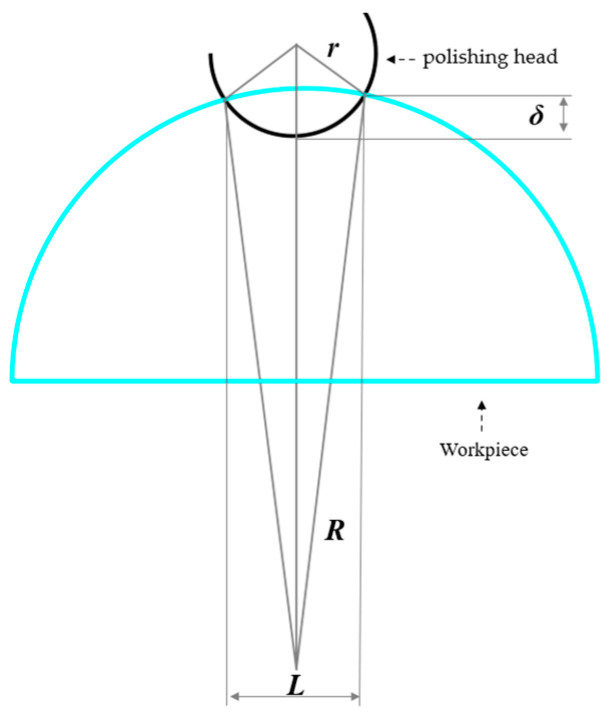
Schematic diagram of the contact between the polishing head and workpiece.

When the radius of the polishing head wheel end is significantly smaller than the radius of curvature of the workpiece, the contact area can be approximated as a circle, simplifying practical engineering issues. As depicted in Figure 8, which illustrates the contact between the polishing head and the aspheric workpiece, the geometric relationship between the two determines the contact width

(35) $$r-\sqrt{r^{2}-{\left(\frac{L}{2}\right)}^{2}}+R-\sqrt{R^{2}-{\left(\frac{L}{2}\right)}^{2}}=\delta$$

The width of contact area *L* is reduced to

(36) $$L=2\sqrt{R^{2}-\frac{{\left[R^{2}-r^{2}+{\left(r+R-\delta \right)}^{2}\right]}^{2}}{4{\left(r+R-\delta \right)}^{2}}}$$

where *r* is the radius of the arc of the wheel end of the polishing head; *R* is the radius of curvature of the workpiece at the contact point; *δ* is the compression of the polishing head; and *L* represents the width of the contact between the polishing head and the workpiece.

### 3.5. Relative Linear Velocity Distribution Model for the Contact Area

**Figure 9:**
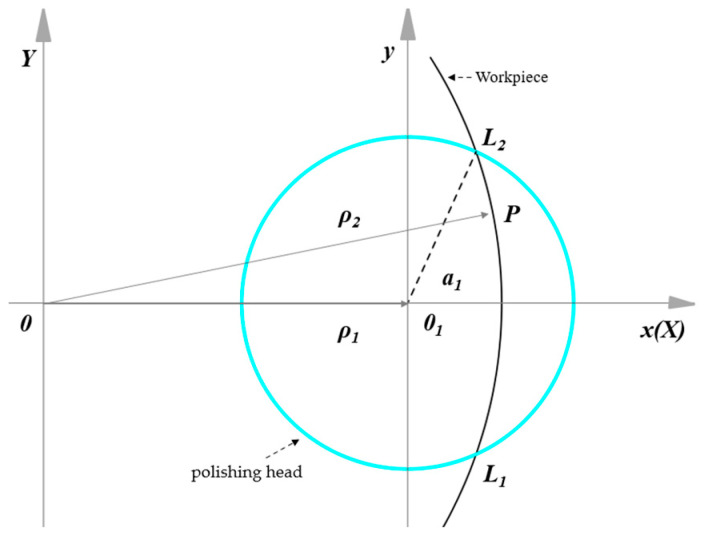
Schematic diagram of the contact area of the polishing trajectory.

According to Preston’s equation, the material removal rate between the polishing head and workpiece is influenced by their relative linear velocity. Adjusting this velocity can enhance polishing efficiency. Figure 9 illustrates the polishing trajectory from the top view, where the polishing head rotates at speed *n_T_*, the workpiece at speed *n_L_*, and their feed speed in the x-direction is significantly smaller, minimally affecting the process.

Assume the center of the workpiece is *O*, the center of the contact area between the polishing head and the workpiece is *O*_1_, and point *P* is any point in the contact area. As the workpiece rotates, point *P* travels an arc length of *l*_1_*l*_2_, with a distance from the radius of the contact area at *O*_1_ to point *P* denoted as *a*_1_. Polar coordinates are more suitable for describing curved motion compared with Cartesian coordinates. Point *O* serves as the pole, *O_x_* as the polar axis, and the polar radius of point *O*_1_ is *ρ*_1_. Furthermore, point *O*_1_ acts as the pole for its own coordinate system, where its polar radius is *ρ*_1_, and for point *P*, its polar radius is *ρ*_2_ with a polar angle *θ*. The distance from point *P* to point *O*_1_ is given by

(37) $$a_{1}=\sqrt{{\left(\rho_{2}\cos\theta -\rho \right)}^{2}+{\left(\rho_{2}\sin\theta \right)}^{2}}$$

The linear velocity of the polishing head at the fixed-point *P* is given as

(38) $$V_{T}=2\pi n_{T}.a_{1}=2\pi n_{T}\sqrt{\rho_{1}^{2}+\rho_{2}^{2}-2\rho_{1}\rho_{2}\cos\theta }$$

The linear velocity of the workpiece at point *P* is given as,

(39) $$V_{L}=2\pi n_{L}.\left|OP\right|=2\pi n_{L}\rho_{2}$$

By combining Equations (37)–(39), we obtain the relative linear velocity of the polishing head and the workpiece at the contact point *P*

(40) $$\begin{array}{l} V=\sqrt{V_{T}^{2}+V_{L}^{2}-2V_{T}V_{L}\frac{\rho_{2}^{2}+{\left(\rho_{2}\cos\theta -\rho_{1}\right)}^{2}-\rho_{1}^{2}}{2\rho_{2}\left(\rho_{2}\cos\theta -\rho_{1}\right)}}\\ =2\pi \sqrt{{\left(n_{T}\rho_{1}\right)}^{2}+{\left(n_{T}+n_{L}\right)}^{2}\rho_{2}^{2}-2\rho_{1}\rho_{2}n_{T}\left(n_{T}+n_{L}\right)\cos\theta } \end{array}$$

## 4. CAM Software Development for Rotational Symmetrical Aspheric Surfaces

### 4.1. Overall Architecture of CAM Software

**Figure 10:**
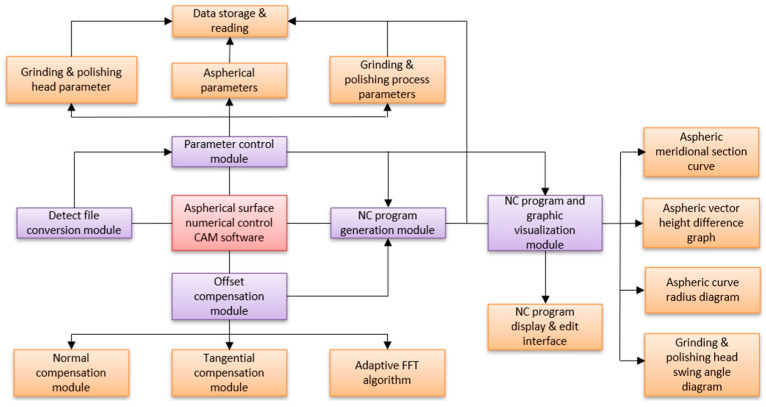
Schematic diagram of the contact area of the polishing trajectory.

The aspheric CNC grinding and polishing CAM software is mainly tailored for Chenna Automation CNC LGS200 grinding and LPS200 polishing processes. Based on software requirements, its various functions are subdivided into several functional modules, forming the foundational units of the system. The overall structure, shown in Figure 10, includes modules such as parameter control, chart visualization, NC program generation and verification, surface shape error correction, and detection data processing.

### 4.2. CAM Software Function Module Design

**Figure 11:**
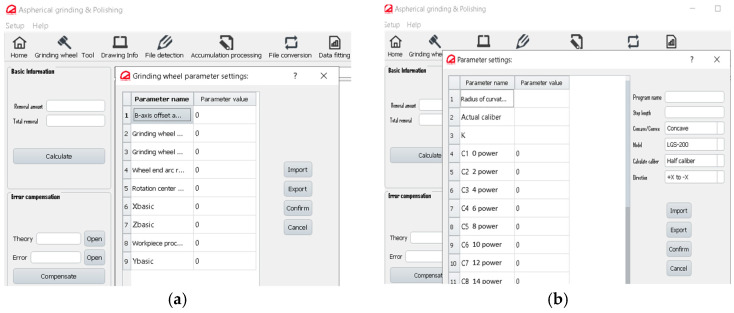
CAM software function module. (**a**) Tool parameter setting. (**b**) Workpiece parameter setting.

The parameter control module consists of the following main parts: (a) tool parameter setting and (b) workpiece processing coordinate setting. In Figure 11a, the tool parameter setting involves handling various parameters. These include polishing tool details like the B-axis offset angle, polishing head length *L_T_*, polishing head middle diameter *D_m_*, wheel end arc radius r, distance from rotary center to hydraulic jaw surface *L_B_*, basic machine tool parameters *X_basic_* and *Z_basic_*. The workpiece processing coordinate setting involves the curvature radius, aperture size, and quadratic coefficient *K*, and higher-order surface coefficients are also managed in this section. It also includes the program name, compensation, concave and convex surface, model, caliber, direction for processing, etc.

## 5. Experimental Verification of CAM Software

**Figure 12:**
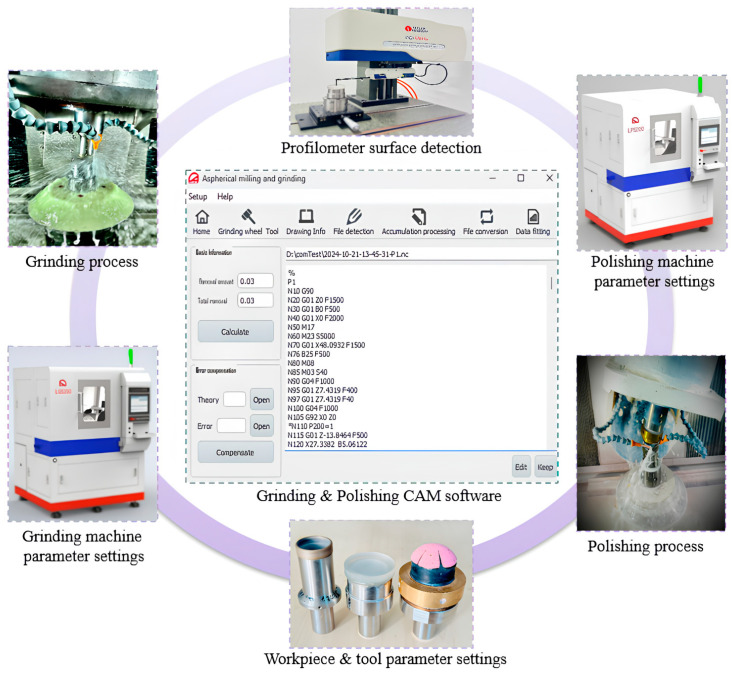
Closed-loop error compensation with CAM software.

To verify the rotational symmetry of the developed CAM software, a closed-loop error compensation study was conducted. The experiment focused on a rotationally symmetrical aspheric optical element with a diameter of 15 mm, made of H-K9L glass. UV glue was applied to the contact area between the workpiece and the tooling, and a dial gauge was used to ensure coaxial alignment. To reduce the thermal effect during grinding and polishing, a water-soluble coolant was used. The detailed setup of the closed-loop experiment is shown in Figure 12, and the parameters for the grinding wheel, polishing tool, and the grinding and polishing processes are provided in Table 1, Table 2, Table 3 and Table 4.

Before conducting the aspheric polishing experiment, the aspheric elements were first ground to achieve the desired aspheric profile using the LGS 200, a three-axis CNC machine. The polishing process was then carried out using the LPS 200, an ultra-precision optical CNC polishing machine. Both machines were made in China, independently developed by Suzhou Chenna Automation Technology Co., Ltd. (Suzhou, China) After each grinding and polishing stage, surface errors were detected using a Taylor-Hobson profiler from the United Kingdom to measure the surface shape error of the aspheric elements.

To verify the closed-loop error compensation process experimentally, we input the basic parameters of the grinding wheel and the aspheric workpiece into the CAM software to generate the initial NC code for grinding. Similarly, the polishing head and workpiece parameters were used to generate the NC code for polishing. The CAM software produced theoretical and actual data files, along with the NC code for both processes. After each grinding operation, surface errors were measured using a profilometer, which generated a .mod file containing the measured error data. The CAM software then converted the .mod file into an Excel format, saving it as the actual measured surface error data. Both the theoretical data from the initial input and the actual measured data were loaded into the CAM software for analysis. The software compensated for the errors and generated new NC codes for the next iteration.

**Figure 13:**
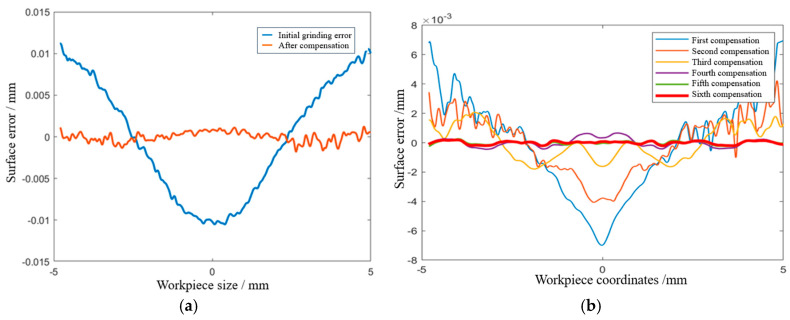
Surface error during grinding and polishing. (**a**) Surface error during grinding. (**b**) Surface error during polishing.

This iterative feedback loop is key to maintaining precision without requiring active control of environmental factors. Even when temperature or vibration introduces small deviations, the CAM software detects and compensates for these errors at each step. Thus, any discrepancies are continuously corrected by generating updated NC codes, ensuring precision over multiple iterations. After completing the grinding phase to remove scratches and irregularities, the same compensation process was applied during polishing to achieve the desired surface finish. This dual-step approach ensured both dimensional accuracy and surface quality, with results from both processes presented in Figure 13.

As shown in Figure 13a, the initial fine grinding of the aspheric surface produced a surface shape error with a PV value of 21.71 μm and an RMS value of 6.99 μm. These relatively high error values indicated the need for surface shape compensation. Using the developed CAM software, a new NC code was generated for this compensation. After applying the correction, the surface shape error was significantly reduced, achieving a PV value of 3.01 μm and an RMS value of 0.59 μm. The error curve flattened considerably, showing that the surface was much closer to the desired shape, with the PV and RMS convergence rates reaching 86.1% and 91.5%, respectively. These results clearly demonstrate effective error convergence, meeting the processing requirements for the aspheric surface and allowing the experiment to proceed to the polishing phase.

In the subsequent polishing process, as shown in Figure 13b, by the sixth error compensation, the surface shape error was further reduced to a PV value of 0.4004 μm and an RMS value of 0.1107 μm. The error curve at this stage was nearly a flat straight line, with the PV convergence rate reaching 97.1%. This indicates a highly effective error convergence, confirming that the polishing process successfully refined the surface to meet stringent optical quality standards.

## 6. Conclusions

This paper presents a novel closed-loop framework for processing rotationally symmetrical aspherical surfaces using optimized material removal functions. Developed on the Qt platform, the CAM software integrates parameter control, chart visualization, sequential NC program generation, surface shape error correction, and detection data processing. The software facilitates seamless feedback between theoretical and measured data, refining NC programs through the entry of polishing parameters and data correction using an Adaptive Fourier Transform Algorithm.

The designed CAM software generates both theoretical predictions and pre-machining NC code based on the input parameters of the cutting tool and workpiece. After machining, the workpiece is measured using a profilometer, and the measured data are integrated back into the software for comparison with theoretical models. This iterative error compensation approach allows the system to generate an optimized NC code for precise surface finishing. Experimental results from grinding and polishing processes confirm that this method effectively controls surface shape errors within the sub-micron accuracy range, demonstrating the software’s reliability and precision.

Unlike previous studies that focus on individual aspects of surface finishing, this research offers a fully integrated closed-loop solution, combining real-time error compensation and adaptive data correction. The iterative feedback mechanism eliminates the need for external tools, enhancing precision while reducing processing time. The use of the Adaptive Fourier Transform Algorithm for data refinement further strengthens the software’s ability to handle complex geometries. This comprehensive approach advances existing surface finishing techniques, offering a streamlined workflow with sub-micron precision. The framework has significant potential for improving the manufacturing of high-precision aspherical optical elements and other advanced optical components.

## Figures and Tables

**Table 1:** Basic parameter of the cylindrical diamond grinding wheel.

Grinding Wheel Parameter	Parameter Value
Grinding wheel length/mm	68.26
Grinding wheel diameter/mm	32.05
Radius of wheel end arc/mm	1.08
Diamond grain size	D20
Diamond concentration/%	80
Binders	Bronze, graphite powder

**Table 2:** Basic parameter of the polishing head.

Polishing Head Parameter	Parameter Value
Polishing head length/mm	70
Polishing head middle diameter/mm	27
Radius of the wheel end arc/mm	23
Polishing film material	Polyurethane polishing film

**Table 3:** Aspheric grinding process parameters.

Process Parameter	Parameter Value
Grinding wheel speed/rpm	3500
Workpiece speed/rpm	40
Grinding wheel feed speed/(m/min)	20
Amount of material removal/mm	0.01

**Table 4:** Aspheric polishing process parameters.

Process Parameter	Parameter Value
Polishing head speed/rpm	3000
Workpiece speed/rpm	20–1000
Feed speed of the polishing head/(m/min)	2–10

## Data Availability

The original contributions presented in this study are included in this article. Further inquiries can be directed to the corresponding author.
